# Evaluation of the Potential Beneficial Effects of *Ferula communis* L. Extract Supplementation in Postmenopausal Discomfort

**DOI:** 10.3390/nu16162651

**Published:** 2024-08-11

**Authors:** Roberta Macrì, Jessica Maiuolo, Federica Scarano, Vincenzo Musolino, Annalisa Fregola, Micaela Gliozzi, Cristina Carresi, Saverio Nucera, Maria Serra, Rosamaria Caminiti, Antonio Cardamone, Anna Rita Coppoletta, Sara Ussia, Giovanna Ritorto, Valeria Mazza, Ezio Bombardelli, Ernesto Palma, Carolina Muscoli, Vincenzo Mollace

**Affiliations:** 1 Pharmacology Laboratory, Institute of Research for Food Safety and Health IRC-FSH, Department of Health Sciences, University Magna Graecia of Catanzaro, 88100 Catanzaro, Italy; federicascar87@gmail.com (F.S.); annalisa.fregola@gmail.com (A.F.); gliozzi@unicz.it (M.G.); saverio.nucera@hotmail.it (S.N.); maria.serra@studenti.unicz.it (M.S.); rosamaria.caminiti@studenti.unicz.it (R.C.); tony.c@outlook.it (A.C.); annarita.coppoletta@libero.it (A.R.C.); saraussia1598@gmail.com (S.U.); giovanna.ritorto@studenti.unicz.it (G.R.); valeria.mazza001@studenti.unicz.it (V.M.); ezio.bombardelli@plantexresearch.it (E.B.); muscoli@unicz.it (C.M.); 2 Laboratory of Pharmaceutical Biology, IRC-FSH Department of Health Sciences, University Magna Graecia of Catanzaro, 88100 Catanzaro, Italy; maiuolo@unicz.it (J.M.); v.musolino@unicz.it (V.M.); 3 Veterinary Pharmacology Laboratory, Institute of Research for Food Safety and Health IRC-FSH, Department of Health Sciences, University Magna Graecia of Catanzaro, 88100 Catanzaro, Italy; carresi@unicz.it (C.C.); palma@unicz.it (E.P.); 4 Renato Dulbecco Institute, 88046 Lamezia Terme, Italy

**Keywords:** menopause, menopausal hormone therapy, phytotherapic compounds, *Ferula communis* L. extract, oxidative stress

## Abstract

Peri-menopausal discomfort can have a detrimental effect on the physical health of women due to physiological and behavioral changes. Estrogen and progesterone-based hormone therapy can alleviate menopausal symptoms, but estrogen supplementation may have negative health effects. The effectiveness of hormone replacement therapy using natural compounds for peri-menopausal disorders is still uncertain. Evidence from in vivo experiments indicates that *Ferula* L. extract in ovariectomized rats leads to better sexual behavior. The effect seems to be linked to the phytoestrogenic properties of ferutinin, the primary bioactive compound in the extract. The purpose of this study was to assess the clinical impact of *Ferula communis* L. extract (titrated at 20% ferutinin, and given at doses of 100 mg/die for 90 days) on the quality of life of 64 menopausal women. The clinical trial was randomized, double-blind, and placebo controlled. Our data showed that *Ferula communis* L. extract reduced by 67 + 9% all symptoms associated to postmenopausal discomfort and enhanced significantly sexual behavior. In addition, the supplement led to a significant improvement of BMI and oxidative stress decrease in the women who received it, while also keeping platelet aggregation within normal levels. Overall, these results could point to the potential use of supplementation with *Ferula communis* L. extract to revert or mitigate menopause dysfunction.

## 1. Introduction

Menopause is a universal biological process that signals the end of a woman’s fertility. Menopause is characterized by the interruption of the menstrual cycle and a decrease in ovarian hormone secretion, typically occurring between the ages of 40 and 50 [1,2]. Various factors, such as geographic location, lifestyle, BMI, and nutrition can influence the age at which women experience menopause [3,4].

A significant number of women experience menopausal symptoms that have a detrimental effect on their physical and mental health. Among these, the following symptoms are particularly recognized: vasomotor (hot flashes, night sweats, and palpitations), cognitive (memory loss and concentration issues), psychological (mood swings, depression, irritability, anxiety, and sleep disorders), atrophic (atrophic vaginitis and bladder irritability), genitourinary tract-related (vulvovaginal atrophy, dyspareunia, sexual dysfunction, stress incontinence, and urinary frequency), alteration of the locomotor apparatus (joint/muscle pain, loss of muscle mass, and loss of bone mass (leading to osteopenia and increased risk of fractures) [5,6,7,8]. The decline in sexual desire and other discomfort-related symptoms are commonly linked to these effects, greatly compromising their quality of life [9]. Hormone therapy during menopause can significantly reduce or eliminate 80 to 90% of symptoms, surpassing other medical interventions.

Specifically, hormone replacement therapy (HRT) involves administering hormones (estrogens and progesterone) and has been FDA-approved to reduce osteoporosis, cardiovascular events, and vaginal atrophy [10]. Over the past few decades, it has become more apparent that estrogen supplements can have detrimental effects on health, and hormone replacement therapy may increase the chances of developing cancer, heart disease, and blood clotting events [11,12,13,14]. The risk of developing cancer, blood clots, strokes, and gallbladder diseases significantly increases with HRT treatment for more than five years [15,16,17,18]. Because of this, a lot of women opt for alternative and complementary therapies to handle menopause symptoms. In the last two decades, there has been an exponential increase in scientists’ interest in plant derivatives due to the myriad advantages these compounds offer in treating dysfunctions involving different organs and tissues [19,20,21,22,23]. In an effort to mitigate adverse effects and improve treatment outcomes, numerous natural compounds have been explored as substitutes or supplements for HRT during menopause [24,25]. Phytoestrogens are plant compounds that mimic estrogen activity and have either an estrogenic or antiestrogenic effect depending on the circulating estrogen levels. The use of phytoestrogens may be advantageous for alleviating menopause symptoms by minimizing the potential side effects that can arise from extended treatment [26]. In recent years, *Ferula communis* L. extract, rich in ferutinin, seems to be of special interest due to its nutraceutical properties and has been suggested as a phytochemical solution to counteract postmenopausal discomfort [27]. The nontoxic chemotype of *Ferula communis* L. has a high percentage of sesquiterpenes such as ferutinin, lapiferin, and teferin. Ferutinin, the primary bioactive compound extracted from various parts of *Ferula communis* L., including roots, leaves, and rhizomes, is responsible for the majority of the effects observed, including phytoestrogenic, antioxidant, anti-inflammatory, antiproliferative, and cytotoxic properties [28,29,30,31,32,33].

Results obtained in different experimental conditions showed important antioxidant and phytoestrogenic regulation with a lack of typical side effects related to estrogenic therapy. The induction of preferential cell death in tumor cell lines by ferutinin indicates its potential as an antineoplastic agent, capable of inhibiting proliferation and causing cell death regardless of estrogen dependency. In particular, an in vitro study showed that *Ferula communis* root extract, thanks to its estrogenic-like properties, may increase the efficacy of tamoxifen for the treatment of hormone receptor-positive breast cancer [34]. Specifically, the combined impact of *Ferula communis* L. root extract allows for the use of lower concentrations of the chemotherapy drug, reducing tamoxifen side effects [34]. Ferutinin shows a distinct ability to induce cytotoxicity specifically in cancer cells at defined concentrations. The results of a comparative study revealed that healthy cells exhibited lower ferutinin toxicity than cancer cells. This finding strengthens the idea that the activity of ferutinin may vary depending on the dose and the type of cell. In fact, a comparative study involving two cancer lines (MCF-7 and Hela) and a normal one (HBL-100) showed lower ferutinin toxicity in healthy cells with respect to cancer cells [34]. This result reinforces the concept that ferutinin activity could be dose- and cell-type dependent. On the basis of this evidence and considering the side effects of anthracyclines on cardiac structure and functionality, a recent in vitro study demonstrated the protective effects of *Ferula communis* L. root extract concentrated in ferutinin (FcFE) on prevention of doxorubicin-induced cardiotoxicity, restoring cell vitality to control levels through the modulation of the cell cycle [35]. In addition, further in vitro studies were performed for the assessment of potential mechanisms which characterize *Ferula*-induced effects in central nervous system (CNS) cells, showing that *Ferula communis* L. extract could protect from lipopolysaccharide-induced neuroinflammation in cultured neurons and oligodendrocytes [29]. Ferutinin has been extensively shown to mimic ovarian activity and may be a viable alternative to HRT with reduced side effects and potential for menopause symptom management [36,37]. With a chemical structure akin to 17-β-estradiol, ferutinin has been identified as a phytoestrogen due to its binding affinity for estrogen receptor subtypes Erα and Erβ [38]. Depending on the cell and tissue type, ERα and ERβ receptors have different effects on transcription factors, eNOS synthesis, cAMP production, ion mobilization, and kinase activation, thereby influencing numerous cellular signaling pathways [39,40,41]. In vitro scientific work has shown that ferutinin has a higher affinity for estrogenic receptor ERα than ERβ [42]. The peculiarity of the sesquiterpene has been confirmed by in vivo studies, and its administration boosts ERα expression. Conversely, ferutinin–estradiol coadministration decreases receptor expression [34]. Ferutinin is a suitable candidate for replacing HRT due to its added benefits. Firstly, unlike estrogen, it can raise the apoptotic index in the luminal and glandular epithelia, preventing excessive cell proliferation and the risk of endometrial cancer [43]. Secondly, ferutinin has been found to favor bone remodeling in rats with estrogenic deficiency similar to menopausal women [37]. Finally, ferutinin enhances the osteogenic differentiation of dental pulp-derived stem cells, leading to higher bone density. This evidence suggests the possible use of ferutinin as a stem-cell therapy modulator to counteract osteoporosis [44]. Conversely, this evidence indicates that ferutinin may serve as a replacement for HRT, leading to significant risk reduction and improved quality of life for menopausal women. Thus, given both the clinical evidence highlighting the crucial role of phytoestrogen to prevent and counteract postmenopausal symptoms [26] and the promising preclinical evidence on the beneficial effects that ferutinin exerts in a dose- and cell-type-dependent manner [34,35,36,37,38,39,40,41,42,43,44], a clinical study was performed to investigate, for the first time, the potential protective action of Ferula L. extract. In particular, a randomized, double-blind, placebo-controlled study was conducted to verify the efficacy and safety profile of a ferutinin-rich Ferula L. extract in women with postmenopausal discomfort.

## 2. Materials and Methods

### 2.1. Ferula communis *L.* Extract and Preparation of Formulation

*Ferula communis* L. extract was kindly provided by Herbal and Antioxidant Derivatives S.r.l. (Bianco, RC, Italy). The chemical composition of the powder extracted from *Ferula communis* L. was determined by high-performance liquid chromatography (HPLC) (by Herbal and Antioxidant Derivatives - H&AD s.r.l.). The chromatographic analysis revealed a ferutinin concentration of 20% in the powder (Supplementary Figure S1 and Table S1).

The instructions for participants were to ingest a 100 mg oral tablet daily for 90 days. In accordance with the European Community Guidelines, all procedures have been carried out regarding dietary supplements.

### 2.2. Clinical Study

A randomized, double-blind, placebo-controlled clinical trial of 64 women with postmenopausal dysfunction was performed. The subjects were enrolled at the IRC-FSH clinical center at the University “Magna Graecia” of Catanzaro from January 2023 to May 2024. All subjects provided written informed consent at the time of the enrollment and the protocol was approved by the local ethics committee (Registry Protocol n. 125, date 24 September 2017; Calabria Region) and met all the principles of the Declaration of Helsinki. The protocol is registered on the ISRCTN registry (ISRCTN78898299).

A starting number of 69 women were selected. The criteria for inclusion were postmenopausal women with a minimum of 12 months of amenorrhea and a follicle-stimulating hormone level above 30 mIU/mL. The criteria involved women with a sexually active life, a stable partner, and postmenopausal sexual dysfunction. The study excluded women with hormonal therapy, diabetes, cognitive disorders, hormone-dependent tumors, psychiatric disease, liver diseases (except prior cholecystectomy), renal disease, cardiovascular disease, and those who used drugs that decrease sexual desire. The women who completed the study (*n* = 64), after an interview and after randomization, were divided into two groups: the placebo group (*n* = 32) received the placebo in a tablet identical in appearance to that used for the second group once a day for 90 days, while the treatment group received *Ferula communis* L. extract (*n* = 32) as an oral tablet (100 mg/die) once a day for 90 days.

The questionnaires used were the Sexual Quotient—Female Version (SQ–F) [45] and the Female Intervention Efficacy Index (FIEI) questionnaire [46].

Developed by Abdo [47], the SQ-F questionnaire includes 10 questions that measure sexual function, exploring aspects such as desire (questions 1, 2, 8), foreplay (question 3), arousal (questions 4, 5), comfort (questions 6, 7), and satisfaction (questions 9, 10). To measure sexual performance, the questions are answered on a Likert scale from 0 to 5. The final score is obtained by summing the points from all questions, where 0 corresponds to never and 5 to always [45].

The FIEI questionnaire [48] involves 7 multiple-choice items. Two questions inquire about side effects and the participant’s sentiments towards joining the study. Four variables of arousal, including vaginal lubrication, sensation, orgasm ability, and sexual satisfaction, are primarily measured by the remaining five questions. In the first five questions, the patient is asked to subjectively assess changes in sexual function after receiving the treatment. Trends were determined using percentages [46].

**Figure 1:**
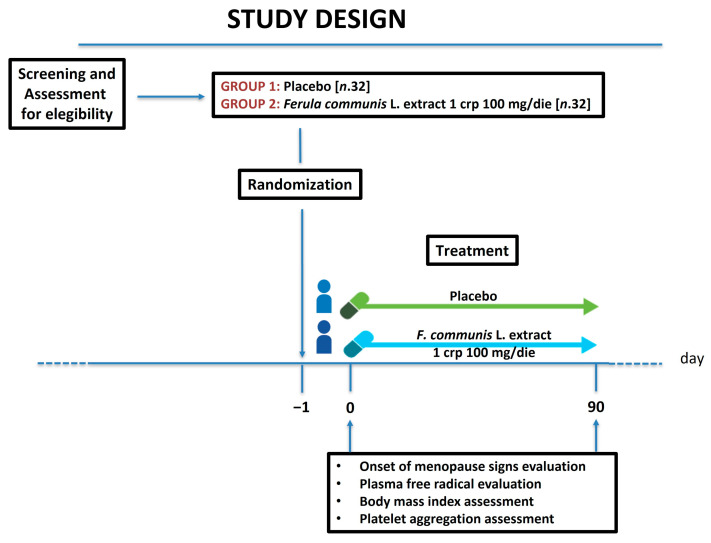
Study design.

The questionnaires were individually administered by the same researcher. The results were examined and explained using the theoretical framework of sociohistorical psychology, which focuses on understanding cultural structure, social organization, and human subjectivity. The data from the initial interview and subsequent visits were compiled and frequencies were allocated to the groups (placebo and *Ferula communis* L. extract) based on the analyzed variables. The onset of menopause sign evaluation and the assessment of plasma free radicals, body mass index, and platelet aggregation were carried out at the baseline and after 90 days of treatment (See Study design, Figure 1).

### 2.3. Plasma Free Radical Evaluation

The measurement of plasma free radicals was conducted as described using a drop of capillary blood from the fingertip. Conventional Carr unit was used to measure plasma free radical concentrations. To assess the oxidative stress status of patients, the study utilized the Free Radical Analytical System (FRAS) (d-ROMs test, cod. MC 003-Diacron, Grosseto, Italy) to measure direct reactive oxygen metabolites. By incubating a small blood sample (20 µL) through the Fenton’s reaction, the d-ROMs test generated alkoxyl and peroxyl radicals from hydroperoxides in a biological sample with the help of iron released from plasma proteins. These radicals can oxidize an alkyl-substituted aromatic amine (A−NH2) in the chromogenic mixture, turning it into a pink-colored derivative ([A−NH2*]+). Oxidative stress status is determined in Carr Units through photometric analysis, with 1 Carr Unit representing 80 µg H2O2/dl. Values above 300 Carr Units suggest oxidative stress [49].

### 2.4. Anthropometric Measurements and Body Mass Index Assessment

The enrolled women were evaluated for overweight, obesity, and changes in body weight using body mass index (BMI) calculations. Body weight and height were assessed for all participants and the measurements were conducted in a fasting state, in an upright position, and without shoes, following standard conditions. The accuracy of measuring body weight and height was 0.1 kg/0.1 cm through a digital Personal Scale (Radwag C315.100/200 OW, Radom, Poland).

The BMI is a statistical index that calculates body fat by considering weight and height, applicable to males and females of any age. In particular, BMI is calculated as weight (kg)/height squared (m^2^). The normal range was established at 18.50–24.99 kg/m^2^. Overweight and obesity are defined as BMI values of 25–29.9 kg/m^2^ and >30 kg/m^2^ respectively [50].

### 2.5. Platelet Aggregation Assessment

Platelet-rich plasma (PRP) samples collected in citrate tubes were assessed for platelet count using an automatic counter (Human Count^®^) and standardized to a mean platelet concentration of 250 × 109 platelets/L. Platelet aggregation was assessed by the inducing agonists collagen and ADP, at concentrations of 2.0 µg/mL and 7.0 µg/mL, respectively (PAP-8e aggregometer—Sentinel Diagnostic, Milano, Italy). For each test, 250 µL of PRP and 250 µL of PPP were used. The aggregation curve was obtained after five minutes of stimulation by ADP and collagen, and aggregation was expressed as a percentage of aggregation calculated through the light transmission in the test solution.

### 2.6. Statistical Analysis

The means of the two groups were compared using various statistical tests including the *t*-test, chi-square test with Yates’s correction, chi-square test, and Mann-Whitney test. In all tests, the significance level was set at 5% (*p* < 0.05). The Wilcoxon Signed Rank test was used to analyze the results obtained through SQ-F and FIEI questionnaires. The analysis was performed using the EPI-INFO program v. 3.3.2 and SPSS software v. 13.0 for Windows.

## 3. Results

### 3.1. Background Information of Women Enrolled in the Study

The women in the two examined groups supplied essential preliminary information for data interpretation. These data are collected and summarized in Table 1.

### 3.2. Results of Sexual Quotient—Female Version Questionnaire

The women participating in this clinical trial were given the Sexual Quotient–Female Version questionnaire both before and after treatment. The results obtained showed a significant improvement of all the domains evaluated (desire, foreplay, arousal, comfort and orgasm) in the *F. communis* L. extract group, after 90 days of treatment.

The results obtained are listed and summarized in Table 2.

The enrolled women underwent additional studies to compare the placebo group and the treatment group (using *Ferula communis* L. extract). The objective was to evaluate the occurrence of menopause symptoms such as hot flashes, vaginal dryness, weight gain, fatigue, insomnia, sweating, irritability, and depression. In this case, the women involved in the randomized, double-blind, placebo-controlled clinical trial were administered the Female Intervention Efficacy Index (FIEI) questionnaire, and the results obtained are listed and summarized in Table 3. All symptoms evaluated were significantly improved following treatment with *Ferula communis* L. plus extract for 90 days.

### 3.3. Antioxidant Power of Ferula communis *L.* Extract

Plasma free radical assessment, investigated by quantifying direct reactive oxygen metabolites using the free radical analytical system (FRAS) on plasma samples of women enrolled in the study, showed a significant modulation of free radicals in *F. communis* L. extract-supplemented subjects in comparison to the placebo group, with an important decrease of oxidative stress (Table 4).

### 3.4. Body Mass Index Assessment Reduction Induced by Supplementation with Ferula communis *L.* Extract

BMI was used to evaluate body weight changes in the enrolled women after 90 days of supplementation. The results obtained showed a significant decrease of BMI within the normal range in women treated with *Ferula communis* L. extract (Table 5).

### 3.5. Ferula communis *L.* Extract Supplementation and Platelet Aggregation

**Figure 2:**
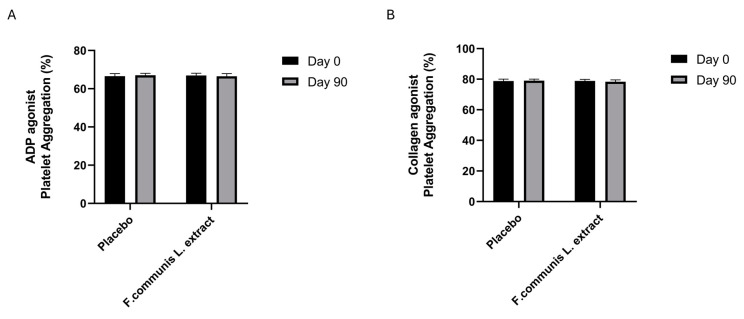
The effects of placebo and *Ferula* L. extract on platelet aggregation in women with perimenopausal discomfort: (**A**) ADP agonist platelet aggregation (%) and (**B**) collagen agonist platelet aggregation (%), respectively. Data are presented as the mean ± standard deviation.

To assess platelet aggregation, the agonists ADP and collagen were used. The median aggregation was in the normal % range at day 0 and after 90 days of treatment, showing that *Ferula communis* L. extract supplementation could prevent the hyperaggregability often induced by hormone therapy in menopausal women (Figure 2A,B).

## 4. Discussion

Sexual behavior is strongly dependent on sex hormones (testosterone and estrogen). In fact, castration in rodents abolishes male sexual behavior, which can be restored following hormonal administration. The binding of sex steroid hormones to their receptors triggers gene expression associated with sexual behavior [51,52]. Evidence has been accumulated in preclinical work showing that *Ferula communis* L. extract (30–60 mg) in ovariectomized rats leads to better sexual behavior. This effect seems to be related to the phytoestrogenic properties of the active compounds present in *Ferula communis* L. extract with a high content of ferutinin [53], although the mechanism of action involved has yet to be further clarified. Evidence suggests that the antioxidant and phytoestrogenic regulation exerted by ferutinin, the principal bioactive compound of *Ferula communis* L. extract, accounts for the lack of common side effects due to estrogenic therapy. Furthermore, the dose-dependent and cell-type-dependent activity of ferutinin gives this extract the ability to counteract different and ambivalent pathological states, shifting from anti-tumor activity at high doses to protective and antioxidant effects of low doses. The protective effects exerted by *Ferula communis* L. extract at low doses on the modulation of the cell cycle in cardiomyocytes exposed to the action of the anthracycline doxorubicin shows the potential of the molecule, which must be further explored in further studies. Phytoestrogenic action and modulation of *Ferula communis* L. extract could play a key role in regulating its action, representing a potential candidate in the fight against menopausal disorders.

Based on the promising protective evidence of the extract, the randomized, double-blind, placebo-controlled clinical trial enrolled 64 women with postmenopausal dysfunction. The women who participated in the study were initially subjected to an interview in order to obtain preliminary information. In particular, all women were over 50 years of age and were in a state of menopause for about two years. Moreover, 89% declared having a partner and carrying out sexual activity. This information (Table 1) was important to determine the presence of menopause and its side effects, as well as any effects on sexual activity [54,55].

### 4.1. Menopausal Discomfort

The Sexual Quotient—Female Version questionnaire was administered to both groups of women involved in the study, before and after treatment with *Ferula communis* L. extract (Table 2). It is interesting to note that the evaluated domains showed a significant improvement following treatment with the extract. According to common belief, menopausal women lose interest in sex, although sexuality remains an important element in women’s lives [56]. The first problem that leads to a reduction in sexual life is the loss of desire, which is estimated to decrease by about 50–55% with the onset of menopause. This effect is hardly explored by women in the medical environment, contrary to what occurs for men. This leads to the emergence of real cultural barriers and personal hardships, resulting in women choosing the simplest option of moving away from sexual life rather than addressing this issue [57]. The reduction in desire also results in a low interest in foreplay and a disruption of orgasms [58]. The decline in estrogenic production during perimenopause and the even lower postmenopausal level are directly associated with these harmful sexual consequences [59]. In fact, in the central nervous system, low concentrations of steroids linked to menopause may result in changes in the activation of certain brain areas responsible for a decrease in sexual arousal [60]. The administration of *Ferula communis* L. extract in this clinical trial significantly improved the domains related to desire, foreplay, comfort, and orgasm after taking the extract for 90 consecutive days. Regarding the symptomatology found within overt menopause, an analogous observation can be made [61]. In particular, the intake of *Ferula communis* L. extract for 90 days led to a statistically significant reduction in flashes, sweating, weight gain, irritability, depression, poor sleep, and vaginal dryness, as shown in Table 3. These data confirm, once again, the results obtained in the in vivo study conducted by Ferretti et al., showing that *Ferula communis* L. extract is able to improve the sexual behavior of ovariectomized rats [53]. The improvement of menopausal discomfort was due to the chemical composition of our extract, determined by HPLC, and revealing a ferutinin concentration of 20%, with ferulenol and teferin at 0.8 and 0.6%, respectively (See Supplementary Figure S1 and Table S1), suggesting that the effect of the extract is mainly due to phytoestrogenic activity of ferutinin. In addition, ferutinin was found to significantly hinder female receptivity in hormone-primed female rats, as observed in in vivo studies. Conversely, teferin and teferdin did not show the same effects, suggesting that ferutinin plays a primary role in the sexual behavior impairment caused by Ferula extract [62].

### 4.2. BMI and Oxidative Stress

Furthermore, there is considerable evidence showing that about 70% of perimenopausal women display overweightness or obesity, associated with a major incidence of morbidity and mortality [63]. Indeed, obesity negatively influences postmenopausal women’s health, leading to an increased risk of cancer [64] and CVD [65]. Obesity is also related to increased oxidative stress [66], since reactive oxygen/nitrogen species (ROS/RNS) generation is caused by an imbalance in antioxidant and oxidant status [67], with the subsequent oxidative modification of several macromolecules including lipids, DNA, and proteins. The alteration of macromolecules and subcellular compartments is in turn responsible for endothelial dysfunctions, thus promoting atherosclerosis and predisposing to CVD [68].

The results obtained in this clinical study confirm the potential protective role of ferutinin to counteract oxidative stress and weight gain in perimenopausal women, showing that *F. communis* L. supplementation was able to ameliorate oxidative status and BMI at 90 days in the subjects enrolled in the study.

Moreover, several data suggest that ROS contributes to atherosclerosis onset and progression, inducing endothelial cell (EC) dysfunction, platelet activation, and vascular remodeling [69].

### 4.3. Oxidative Stress and Platelet Aggregation

ROS/RNS, produced in an unbalanced manner following hormonal changes due to menopause, could interact with platelets through the ADP receptor, triggering the same intracellular signaling cascade. In addition, nitrogen peroxide induces fibrinogen activation and fibrin clot stabilization. Indeed, the pro-oxidant state leads to endothelial dysfunction and platelet activation in primary hemostasis, which converge and amplify, thus producing severe prothrombotic effects [70]. In particular, in human dysfunctions which are associated with high cardiovascular risk, including obesity, Ox-LDLs are able to induce platelet activation through CD36 binding, and 8-epi-PGF_2α_ leads to platelet aggregation via thromboxane (TX) A_2_ receptors (TP), releasing adenosine diphosphate (ADP) [71].

An interesting piece of evidence which requires further investigation concerns the potential ability of the extract, due to its antioxidant and phytoestrogenic properties, to improve the overall dysfunctional state of perimenopausal women without, however, altering the platelet aggregability.

The main limitation of our study consists in the small number of subjects enrolled. Generalizing the results of our study is not possible because it was carried out in a single center. There is a need for multicenter clinical trials involving a larger cohort of patients. Furthermore, osteoporosis, a significant menopause-related risk factor, was not assessed due to the need for a longer period of management and observation.

## 5. Conclusions

In conclusion, the data that we have obtained in the clinical study suggest that *Ferula communis* L. extract can be considered an excellent candidate for the treatment of symptoms during menopause and could bring about decisive improvements in menopausal dysfunction. However, further confirmatory experiments should be carried out, examining a larger cohort of patients and for a longer time range of treatment, taking into consideration various further aspects including the effects in different organs and tissues, which could influence the supplementation. Furthermore, a combination and association of the extract with conventional therapy could be tested through preliminary preclinical studies to be subsequently translated into clinical trials, trying to increase the beneficial actions while at the same time reducing the potential harmful effects.

## Figures and Tables

**Table 1:** Clinical and demographic characteristics of menopausal women in the placebo group and the *Ferula communis* L. extract group.

Characteristics	Placebo (*n* = 32)	*F. communis* L. Extract (*n* = 32)
Age (years)	52 ± 4.3	52 ± 4.8
Body Weight (kg)	68.5 ± 8	66.7 ± 9
Height (cm)	164 ± 10	165 ± 8
Menarche	12.5 ± 1.5	12.5 ± 1.2
Menopausal age (years)	50 ± 4.2	50 ± 4.5
Blood Pressure (mmHg) Systolic Diastolic	118 ± 12 70 ± 11	119 ± 13 70 ± 12
Years since menopause	>1	>1
Educational level High school degree University degree	50% 50%	50% 50%
Civil status: Married Single	89.2% 11.8%	91.2% 9.8%
Race: White	100%	100%

**Table 2:** Domains evaluated according to the Sexual Quotient—Female Version questionnaire before and after treatment (placebo group and *Ferula communis* L. extract group). Data are presented as the mean ± standard deviation. The *p*-values refer to the outcome of Wilcoxon Signed Rank test; ***: *p* < 0.001 placebo vs. *Ferula communis* L. extract group.

Domains Evaluated	Placebo (*n* = 32)		*F. communis* L. Extract (*n* = 32)	
	Before	After	Before	After
Desire	6.2 ± 2.4	6.4 ± 2.8	6.4 ± 2.3	9.2 ± 2.5 ***
Foreplay	2.4 ± 1.6	3.1 ± 1.6	2.9 ± 1.5	4.2 ± 1.0 ***
Arousal	5.4 ± 2.1	5.2 ± 2.5	4.4 ± 2.1	7.5 ± 2.1 ***
Comfort	6.0 ± 2.1	6.4 ± 2.1	5.9 ± 2.3	8.4 ± 1.6 ***
Orgasm	5.0 ± 2.4	5.4 ± 2.8	4.6 ± 2.1	6.9 ± 2.1 ***

**Table 3:** The effects of placebo and *Ferula communis* L. extract on flashes, sweating, weight gain, irritability, poor sleep, and depression in women with perimenopausal discomfort. Data are presented as the mean ± standard deviation. The *p*-values refer to the outcome of Wilcoxon Signed Rank test; ***: *p* < 0.001 placebo vs. *Ferula communis* L. extract.

Symptoms Evaluated (% among Women Entering the Study)	Placebo (*n*=32)		*F. communis* L. Extract (*n*=32)	
	Before	After	Before	After
Flashes	88 ± 4	82 ± 5	89 ± 3	21 ± 3 ***
Sweating	86 ± 3	84 ± 4	85 ± 4	32 ± 4 ***
Weight gain	68 ± 3	63 ± 4	66 ± 5	45 ± 4 ***
Irritability	89 ± 5	80 ± 4	88 ± 4	24 ± 3 ***
Depression	92 ± 4	85 ± 5	93 ± 3	22 ± 3 ***
Poor sleep	96 ± 4	82 ± 3	94 ± 4	32 ± 5 ***
Vaginal dryness	94 ± 3	92 ± 4	92 ± 5	26 ± 4 ***

**Table 4:** The effects of placebo and *Ferula communis* L. extract on oxidative stress in women with perimenopausal discomfort. Data are presented as the mean ± standard deviation. The *p*-values refer to the outcome of Student’s *t*-test; *: *p* < 0.05 placebo vs. *Ferula communis* L. extract.

Domains Evaluated	Placebo (*n* = 32)		*Ferula communis* L. Extract (*n* = 32)	
	Before	After	Before	After
Oxidative Stress (U/Carr)	375 ± 18	358 ± 14	379 ± 11	328± 16 *

**Table 5:** The effects of placebo and *Ferula communis* L. extract on BMI in women with perimenopausal discomfort. Data are presented as the mean ± standard deviation. The *p*-values refer to the outcome of Student’s *t*-test; *: *p* < 0.05 placebo vs. *Ferula communis* L. extract.

Domains Evaluated	Placebo (*n* = 32)		*Ferula communis* L. Extract (*n* = 32)	
	Before	After	Before	After
BMI (kg/m^2^)	25.2 ± 1.1	24.9 ± 1	25.1 ± 0.9	23.1± 1.1 *

## Data Availability

The data presented in this study are available upon request from the corresponding author.

## Supplementary Materials

The following supporting information can be downloaded at: https://www.mdpi.com/article/10.3390/nu16162651/s1, Figure S1 and Table S1: HPLC analysis.

## List of Abbreviations

Body mass index	(BMI)
Cardiovascular Disease	(CVD)
Central Nervous System	(CNS)
Cyclic adenosine monophosphate	(cAMP)
Estrogen receptor alpha	(Erα)
Estrogen receptor alpha	(Erβ)
Female Intervention Efficacy Index	(FIEI)
Fetal bovine serum	(FBS)
Food and Drug Administration	(FDA)
Hormone replacement therapy	(HRT)
Quality of life	(QOL)
Sexual Quotient—Female Version	(SQ–F)
