# Supplementary material for: Evaluation of the Potential Beneficial Effects of Ferula communis L. Extract Supplementation in Postmenopausal Discomfort
Source: Nutrients. 2024 Aug 11;16(16):2651. doi: 10.3390/nu16162651 (PMC11357168; doi:10.3390/nu16162651)
Supplement: Supplementary file 1 [file nutrients-16-02651-s001.zip › nutrients-3134577-supplementary.pdf]

Supplementary S1

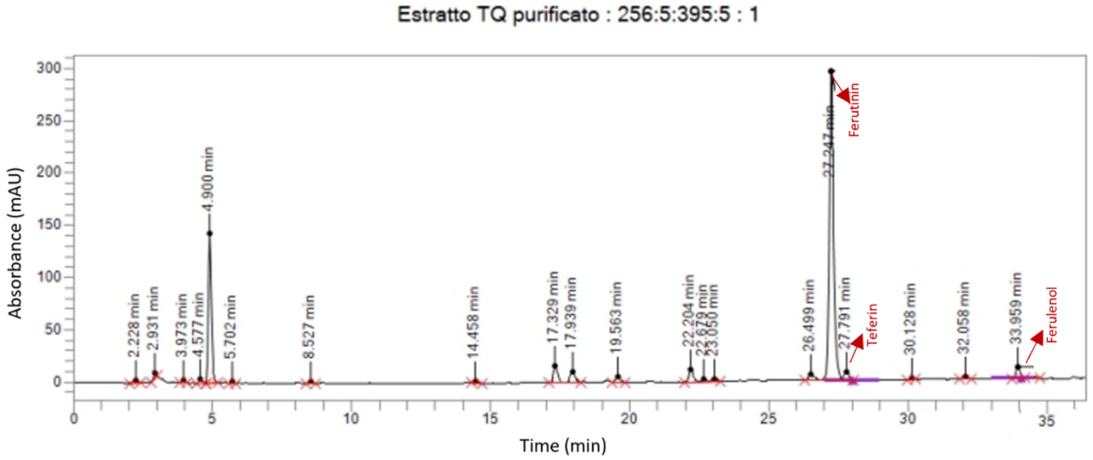

**Component name**	**Time (min)**	**Area**	**Final Amount (ppm)**
Ferutinin	27.247	3,230,690	205,000
Ferulenol	33.959	101,320	8,002
Teferin	27.791	90,223	6,380

Supplementary Figure: HPLC spectrum of Ferula extract, in which ferutinin is present at concentration of 20% in the extract; however, ferulenol and teferin are present at concentration < 1%.
